# Acknowledgment to the Reviewers of *Journal of Functional Biomaterials* in 2022

**DOI:** 10.3390/jfb14020054

**Published:** 2023-01-19

**Authors:** Journal of Functional Biomaterials Editorial Office

**Affiliations:** MDPI AG, St. Alban-Anlage 66, 4052 Basel, Switzerland

High-quality academic publishing is built on rigorous peer review. *Journal of Functional Biomaterials* was able to uphold its high standards for published papers due to the outstanding efforts of our reviewers. Thanks to the efforts of our reviewers in 2022, the median time to first decision was 13 days and the median time to publication was 36 days. Regardless of whether the articles they examined were ultimately published, the editors would like to express their appreciation and thank the following reviewers for the time and dedication that they have shown *Journal of Functional Biomaterials*:
Abbas RahdarŁukasz ZapałaAbdollah AllahverdiLuminiţa Maria NicaAbdolreza JamilianM. Azizur RahmanAbdul Azeez Abdu AliyuM. R. M. AsyrafAbdul WadoodMadalina PandeleAbdulkarim AmirovMagdalena GacaAbul Kalam AzadMagdalena GłąbAdil AbdullahMahesh P. BhatAdnan AynaMahmoud GhomiAdrian FifereMajid MalboubiAdriana BigiMakoto HasegawaAgam BansalMalgorzata Zakłos-SzydaAgata Jabłońska-TrypućManendra Babu LankadasariAgnieszka Gadomska-GajadhurManisekaran RavichandranAgnieszka KyziołManmohan SinghalAhmed El-FiqiManuel GraçaAhmed FatimiManuel Marques FerreiraAidin Bordbar-KhiabaniMaosud Soroush BathaeiAike QiaoMarc BohnerAjay Vikram SinghMarcello BertoAjinkya PawarMarcelo GiovanelaAlberto Adanero VelascoMarcin KozakiewiczAlberto Rodríguez-ArchillaMarcin WekwejtAlejandro J. AlvarezMarcio Mateus BelotiAlena Opalkova SiskovaMarco C. BottinoAlessandra BiancoMarco GovoniAlessandro GiudiciMarco LelliAlessandro NotaMarco ParenteAlessia FamengoMarco PortelliAlex DommannMarco TallaricoAlex KuchumovMarco TatulloAlexander FominMarcos BrioschiAlexander I. TitkovMarek BrzezinskiAlexander KomissarovMarek WisniewskiAlexandr Alexandrovich KolobovMargarita A. KhimichAlexandros K. NikolaidisMargit SchulzeAlexandru BurlacuMaria Angeles HerranzAlexandru VlasaMaría Ángeles Sánchez-GarcésAlexey A. IvanovMaria Annunziata CarluccioAlfredo IandoloMaria AntoniadouAlfredo RoncaMaria BarbolinaAli ArabMaria Cristina TanziAlperen DegirmenciMaria LavlinskayaAlvin Kai-Xing LeeMaria LazaridouAmal NarayananMaria NikolovaAmeeduzzafar ZafarMariana BusilaAmit Md Estiaque ArefinMarija MojicevicAna I. FernandesMario RaspantiAna IvaniševićMarioara AvramAna M. BeltranMark B. FramptonAna M. Carmona-RibeiroMárk FráterAna Teresa Corte-RealMarta SzekalskaAnca DinischiotuMartin MichálekAnca MazareMarwa EmamAnca PetranMasrina Binti Mohd NadzirAndrea CochisMassimiliano De PaolisAndrea FinMassimo CarossaAndrew MakanyaMatej ParAndrey E. KrauklisMatheus PolettoAndrey V. BorodaMatteo Bruno LodiAndrzej BrudnickiMatteo ZanoccoAndrzej PuszkaMatthew Gavino DonaduAndy Wai Kan YeungMatthias NeesAngel Josabad Alonso-CastroMauricio A. Sarabia-VallejosÁngel Serrano-ArocaMautusi MitraAngela De BonisMax MenziesAngela SpoialăMaximilian HammerAngelos SikalidisMazeyar Parvinzadeh GashtiAnil TharappelMd Habban AkhterAnisoara CimpeanMd. Nizam UddinAnkit GangradeMehrez E. El-NagarAnna BelcarzMeysam KeshavarzAnna KazachenkoMh Busra FauziAnna KostyukMichael B. FischerAnna MichopoulouMichael D. GrayAnna Morawska-ChochółMichael EllisonAnton ManakhovMichał PikułaAnton S. KonopatskyMichele FioreAntonia ResslerMieszko WieckiewiczAntonija TadinMiguel Gisbert-GarzaránAntonino PollicinoMiguel MaureiraAntónio GinjeiraMihaela DinuAntonio GloriaMihaela HedeșiuAntonio Mario LocciMihai AxinteAntonio ScaranoMikhail KryuchkovAntonios AnastasiouMikołaj DąbrowskiAnuraag BoddupalliMilica LukićAnuradha PrakkiMing-Tzu TsaiAnying SongMing-Yu LiArgiris KolokithasMin-Huey ChenAris GiannakasMirko ManettiArjun Prasad TiwariMirko MaturiArkadiusz SzarekMirona Palczewska-KomsaAron LazaryMiroslav PohankaArpita RoyMirza Rustum BaigArturo Sanchez-PerezMoataz ElgezawiArun KumarMohammad AlamAshkan BighamMohammad ArefiAshok K. ShakyaMohammad AshfaqAssunta PandolfiMohammad Azam AnsariAstgik PetrosyanMohammad HassanAtanu ChakrabortyMohammad Javad AmiriAtul BabbarMohammad Khursheed AlamAurelian Sorin PascaMohammad Tajul IslamAyako WashioMohammad Z. KabirAydin Bordbar-KhiabaniMohammed A. TahaAyoub MoghadamMohammed F. HamzaAzadeh AsefnejadMohammed GrawishAzadeh NilghazMohammed H. MohammedAzhwar RaghunathMohammed HawashBabak ChehroudiMohammed SayedBansi MalhortaMohd Abul KalamBarbara JadachMohmed Isaqali KarobariBarbara ŁapińskaMona Aly AbbassyBarbara SartoriMonica Mattioli Belmonte CimaBart PijlsMonica YamautiBeata Kalska-SzostkoMonika FurkoBeata Morak-MłodawskaMonika JenkoBenny RyplidaMoniruddoza AshirBernd W. SiguschMorgan HamonBishweshwar PantMorteza Fallah KarkanBjörn RathMostafa SeifanBogdan IstrateMousumi DebnathBoris KozlovMuhammad AyyoobBruce MilthorpeMuhammad Imam AmmarullahBruno ChrcanovicMuhammad Imran RahimBudimir MijovićMuhammad IqhrammullahBwalya WitikaMuhammad SaleemByeong-Ui MoonMuhammad Sohail ZafarC. K. AbdullahMunirah Binti Sha'BanCândida Teixeira TomazMunteanu CorneliuCanel EkeMuthaiah ShellaiahCanran WangMyeong-Hyeon WangCanzeng LiangMyoung-Ryul OkCarina BalcoşNafisa GullCarina CruchoNagavendra KommineniCarles FalconNaji KharoufCarlo BizNaozumi TeramotoCarlo DiaferiaNaresh KasojuCarlos CaroNaresh KumarCarlos Cuevas-SuárezNassar MohannadCarlos E. NemcovskyNatallia DubashynskayaCarlos Fernando MourãoNavaneeth Krishna Rajeeva PandianCarlos Martín ArdilaNectarios VidakisCarlos RovereNehal ElsherbinyCaroline MoraesNenad IgnjatovicCatarina RosadoNermeen YosriCatarina SeabraNhung H. A. NguyenCátia DominguesNibedita LenkaChang LiuNicholas FischerChao QiNicolas Heinz Von Der HoehChaobo HuangNikolett Kállai-SzabóChara SimitziNina G. AttikChe Azurahanim Che AbdullahNitin KhandelwalCheng Ee MengNitin SharmaCheong-Weon ChoNittiya SuwannasomChe-Yu LinNobuyuki FutaiChi Bum AhnNoriko HiraishiChiara BernardiniNuray AcarChiara PagliucaOana CadarChinmay SatamOctavian AndronicChinmaya MahapatraOla A. TarawnehChin-Yu LinOleg ShichalinChiranjeevi KorupalliOleg V. GradovChuda ChittasuphoOlena IvashchenkoChun Hung ChuOlga V. Frank-KamenetskayaChung-Kwei LinOlukayode Samuel AkinwamideChunxia LiOmid NabinejadClaudia-Geanina WatzOrlando Aguirre GuedesConstantin Von SeeOscar Gil-CastellConsuelo CelestiOskar SachenkovCorrado PiconiOvidiu Cristian OpreaCristian ScheauOzlem Ipek Kalaoglu-AltanCristina QuílezPankaj DhatrakCsaba BalázsiPankaj VadgamaDagnija LocaPaolo CapparèDaniel B. YaroshParbeen SinghDaniel BaeckerPatrick HaubruckDaniel Fernández-QuirozPaula Isabel SoaresDaniel RealPaulina KazimierczakDaniela BuchaimPaulo J. PalmaDaniela CalinaPavel BradnaDaniela Cristina CulitaPavel PadnyaDaniela MontiPavel RehakDaniele LandiPavol FarkašDanimir JevremovićPaweł RamosDanko BakarčićPaweł TurekDapeng WangPayal GangulyDario Di NardoPayam ZahediDariusz GóralPeng ChenDariusz JarzabekPeng YuDawid ŁysikPengyu HongDebanjana GhoshPetra MaierDeepak Kumar KhajuriaPeyman GholamiDenis GirardPhilippe HujoelDenis V. VoroninPierre WeissDenise BellisarioPierre-Yves Collart-DutilleulDhaval ChauhanPietro AusielloDhruvajyoti RoyPilaiwanwadee HutamekalinDiana CiolacuPing LiDiana G. Zarate-TriviñoPiotr KurcokDiana PortanPiotr MorasiewiczDiego AntonioliPiotr PiszczekDiego QuirogaPiotr WychowańskiDimitrios BikiarisPoulomi SenguptaDimitrios DionysopoulosPradeep KumarDiptonil BanerjeePranothi MulintiDivya SharmaPrashanth RavishankarDomagoj GlavinaQi GuDong-Wook HanQi XiangDorin GheorgheQian YangDuarte MarquesQiwen QiuEbrahimi MehdiQuanguo HeEdina RusenRabia Sannam KhanEdward RenRachmat HidayatEdwin-Joffrey CourtialRafal RakoczyEgor OsidakRaffaella De PaceEkaterina O. MikhailovaRahul Madathiparambil VisalakshanElena SvirshchevskayaRaluca IanchisElena V. ProskurninaRamesh ParameswaranEleonore FröhlichRamona CimpoeşuElia BariRangasamy JayakumarElisa BelluzziRanjith Kumar RajamaniElisa BoaniniRebeka RudolfÉlton Gonçalves Onçalves ZenóbioRegina Maria Puppin-RontaniElżbieta TrafnyReka BarabasEmil N. SobolRenata WłodarczykEnrico BrugneraRicardo Mallavia MarinEnrico GherloneRiccardo BeltramiEnrico LeoniRim BourgiEnsanya Ali Abou-NeelRita GelliEri Miura-FujiwaraRizwan WahabErlin ZhangRobert GuidoinErnesto Beltrán-PartidaRoberta GasparroErshuai ZhangRoberto GristinaEsam YahyaRoberto TeghilEsmaeel SharifiRocco FrancoEsperanza DíazRocco PapaliaEssam Al-MoraissiRodolfo RedaEugenio Velasco OrtegaRodrigo FrançaEunice CarrilhoRodrigo ResendeEvangelia VouvoudiRogério Leone BuchaimEvangelos P. HadjigeorgiouRoland KaunasEvgenia Korzhikova-VlakhRoman A. SurmenevEwa StodolakRoman DeevEwelina PiątczakRoman SurmenevFabian ItelRongguo RenFabian ZanellaRosemeyre Amaral CordeiroFabiano RodembuschRossana IzzettiFaiyaz ShakeelRoxana LucaciuFang-Cheng LiangRuben Panadero AgustinFarid MenaaRuchi RoyFarzaneh SabbaghRupendra ShresthaFatma Turna DemirRusso EleonoraFausto ZampariniRyan M. Van HornFederica GiordanoSabrina BelbekhoucheFei WangSabrina EhnertFei XingSachit AnandFei XuSaeid KargozarFelice FemianoSagar AryaFeng ChenSagar RoyFlávio Alberto Da Silva FigueiraSajjad ShiraziFlavio De Alcantara CamejoSalber JochenFlorent MorfoisseSalvatore GalloFlorin MiculescuSalvatore ScolavinoFong-Yu ChengSameer AwadFrancesca DiomedeSanaa Najeh Al-Haj AliFrancesco InchingoloSanchita KunduFrancesco Saverio LudovichettiSandeep Kumar Reddy AdenaFrancisca García-Moreno NisaSandra Kalil BussadoriFrancisca RodriguesSandra PinaFrancisco Humberto Xavier-JuniorSang-In ParkFrederic HeimSangram SamalFrederic MeyerSantiago P. AubourgFulden Ulucan KarnakSara BernardiG. Devanand VenkatasubbuSara C. ZapicoGabriela BiliutaSara MoradiGaetano PaoloneSara TargońskaGalina A. DavidovaSarah PixleyGang ChengSaravanan MuthupandianGaofeng QuanSarbaranjan PariaGennaro RuggieroSarhang HamaGeoffrey MitchellSatoshi KomasaGeorge Alexandru MafteiSaturnino Marco LupiGerardo FatoneSébastien PenninckxGhasem SargaziSeigo OhbaGigliola LusvardiSelestina GorgievaGionata FragomeniSelmin FrancescaGiovanna De LucaSelvaraj Rajesh KumarGiovanni MergoniSema BelliGiuseppe Lo GiudiceSenka MeštrovićGiuseppe MaccagnanoSergei KulinichGiuseppe MinerviniSergey AnikeevGiuseppe VarvaraSergio MazzoleniGo AnanŜerife Pınar YalçınGoucheng WangSerkan YílmazGregori M. KurtzmanSeyed Morteza NaghibGrzegorz AdamekSeyyed Mojtaba MousaviGrzegorz JanowskiShakeel Ahmad KhanGrzegorz MatulaSharafaldin Al-MusawiGrzegorz SkrabalakShayfull Zamree Abd RahimGuillermina BurilloShazid Md. SharkerGustavo FernandesShepard HurwitzGustavo Filipe PintoShih-Chung WeiGutemberg AlvesShih-Feng ChouGuya Diletta MarconiShilpa BhandiHadi KordestaniShokouh AttarilarHadi RastinShu-Fen ChuangHafiz Muhammad Salman AjmalSiddhartha PatiHagen FrickmannSigmar SchnutenhausHaiyang A. YuSimin SharifiHamedreza JavadianSimon FoxHamid Bakhsheshi-RadSimona BadilescuHamid Reza Bakhsheshi-RadSinan EğriHanna LewandowskaSiu Hong Dexter WongHans-Christian SiebertSławomir KuleszaHasan Zuhudi AbdullahSnehashis PalHassan Al-KaragolySnejana BakardjievaHassan M. A. HassanSnezana Ilic-StojanovicHassan M. IbrahimSoheil Abbaspour-RavasjaniHåvard J. HaugenSolehuddin ShuibHeithem Ben AmaraSónia CarabineiroHelena MartynowiczSorout ShaliniHend Abdel BarSoumen DasHer-Hsiung HuangSowmya SrinivasanHervé ReychlerSpundana MallaHimanshu KathuriaStefano PaganoHonghao HouStefano RavaioliHornyák IstvánStefanos KikionisHossein Maleki-GhalehStephane BolducHuifen ChenSteve ElderHuiliang CaoSteven PrawerHung-Yin LinSudhir ShendeHusam YounesSudipta PanjaHyo-Eon JinSulayman OladepoIgor BuzalewiczSumeet Kumar SinghIhor S. VirtSumit Sinha RayIka AnaSunghoon ShinIk-Hwan KimSung-Woon OnIleana IeloSuresh K. VermaInderbir SinghSuresh SagadevanInes DespotovicSuryadi IsmadjiIoan PeteanSusana R. SousaIoan StamatinSusanna FerrarioIoana ChiulanSusanna PilusoIoannis KaroussisSuzana FilipovicIolanda De MarcoSven RutkowskiIonut LuchianSvetlana AnticIonut-Cristian RaduSvetlana BatashevaIrmanida BatubaraSvetlana BuyakovaIstván CsarnovicsSvetlana SaikovaIuliana-Mihaela DeleanuSwee Leong SingIvan LesovSybille HasseIvan TorresSyed NabilIvica PelivanSylwia ŁaganIzabela Młynarczuk-BiałyT. HanasJaafaru Mohammed SaniTae Hoon KongJacek SawickiTaichi TenkumoJacobo Hernández-MontelongoTakaaki InoueJagoda PlaczkiewiczTamás SoványJakub HadzikTanja Grubić KezeleJakub MatusiakTanja ZidaričJames ChowTao ChenJames Kit-hon TsoiTao FuJames V. AquavellaTapas MandalJanja TrcekTarek MohamedJános KissTatsuya MorimotoJarosław ŻmudzkiTejas BarotJarosław ZubrzyckiTeng ChiJasminka TalapkoTeresa RussoJaved AhmadTereza KauerovaJayachandran VenkatesanThaigarajan ParumasivamJaysukh H. MarknaThibault GallavardinJeevithan ElangoThomas GoudoulasJefferson MattosThomas PatersonJen-Chang YangTibor SopčákJenni VirtanenTihomir VasilevJesús Martín-GilTimur Sh. AtabaevJesús-María García-MartínezTina McKayJialei LiuTiziano TestoriJianhua ZhangTomasz KaczmarzykJiashen LiTomasz KlekielJiawei WangTomasz KoziorJie HuangTomasz PanczykJin-Chul KimTomokazu HattoriJinlian HuTomoyuki IwataJinrong MaToshiharu ShinokaJiri SmilekToshiki MiyazakiJithin VishnuTsanka D. DikovaJizhe CaiTsanka DikovaJoan Manuel Rodríguez-DíazTsung-Jen WangJoanna CzechowskaTsung-Li LinJoanna KolmasTsuyoshi KimuraJoanna MystkowskaUlrike RitzJoanna WezgowiecUmberto TarantinoJoão CaramêsUrban BrenJoão CondeUrszula BazylinskaJoão M. SantosVadim ElaginJoão Paulo LealValentina Di NisioJohn DzimianskiValeriy SkryshevskyJohn JosephVan Giau VoJohnson V. JohnVanesa EstebanJonghyun OhVasilica PopescuJong-Tae ParkVasudev BallalJorge MartinsVenkatakrishnan RengarajanJosé Antonio Morales-GonzálezVi-Khanh TruongJosipa VlainićViktoria RajtukovaJozefina KatićVincent SallesJuliana FernandesVincent YeungJuliane Salbach-HirschVincenzo ToscoJunaidi KhotibVineeth M. VijayanJun-Kyu ParkVinita VishwakarmaJunxi WuViorel PaleuJunxia LuViorica Railean-PlugaruJunzi WuVirender SinghJustyna SulejVirginija JankauskaiteK. M. Faridul HasanVishnu Priya MuraliKaan OrhanVishnu RevuriKadir OzaltinVito Domenico BrunoKalyan RameshVitor CarneiroKamil GareevVivek P. ChavdaKamol DeyVivian De WaardKannan ChidambaramViviana VergaroKapil PatelVladimir MuronetzKarekin EsmerianVu Hoang Giang PhanKarol Alí Apaza AlccayhuamanWaldemar MrózKarthika PrasadWei ChenKatarzyna JelonekWei FeiKatarzyna KrukiewiczWei Long NgKatarzyna Sykłowska-BaranekWei WuKatarzyna SzałapataWei-Dong HeKatarzyna SzepietowskaWeidong YangKatarzyna TandeckaWeifeng LinKaushik PalWen ZhangKeke WuWenbo BuKelvin FoongWen-Cheng ChenKelvin Ian AfrashtehfarWen-Fu HoKensuke KumeWen-Hua ZhengKesawat Mahipal SinghWenjie ZhouKeskanya SubbalekhaWissam FarhatKevin WillyWon-Bae ParkKiho ChoXiaohong WangKinga K. TurzóXiao-Kun OuyangKishore DebnathXiaoliang QiKlara MagyariXiaoning ZhangKlaus GratzXiaoyu HuangKlemen BohincXiaoyu NiuKoichi ShinkaiXin LiuKonstantin Johannes ScholzXinping ZhangKosuke NozakiXuan MeiKotai LaszloXuesong LiKrishan K. VermaYamin LiKristina LozieneYan LiKrzysztof MazurekYan WangKuofeng HungYannis DimakopoulosLaetitia ChauviereYanxiao LiLamprini MalletzidouYao ZhaoLatif SumeraYaodong LiuLaura CatenacciYau Kei ChanLauren ButaricYe ZhouLaurence J. WalshYi ZhouLavinia ArdeleanYichao ZhaoLavinia Cosmina ArdeleanYifeng CaoLe YuYin Chai WangLech B. DobrzańskiYing SunLee SherryYing-Guo ZhouLee Te ChuanYingming YangLei HouYit Lung KhungLeonardo RestaYogendra Pratap SinghLeopoldo FornerYolanda Martínez-BeneytoLeto-Aikaterini TzivelekaYong JinLi-Ching ChangYongbo LuLidia GavicYongkui LiLiguo QinYoshikatsu KogaLihong SuYoung-Hun JeongLiliana Argueta-FigueroaYu-Hsiang KuanLing YuYuh‐Yih LinLionel GamarraYuki NagamatsuLiron Limor IsraelYupeng ChenLiudmila D. IskhakovaYuqian ZhangLiviu DutaYuqin QiaoLoredana TammaroYuriy F. ZuevLorenzo Degli EspostiYuwei FanĽubomír MedveckýYuyan JiangLuca BoselliZehra EdisLuca CevolaniZhaozhu ZhengLuca GiachettiZhong LiLuca PezzatoZhuang LiLuca TestarelliZhuoran MaLucia CatucciZihao OuLucian Toma CiocanZohaib KhurshidLucilene Delazari Dos SantosZois TsinasLuis Alberto Gaitán-CepedaZorana MilanovicLuis Andrés López FernándezZorka Z. VasiljevicLuísa FialhoZufu LuLukas PfeiferZuolin WangŁukasz JohnZuzanna J. KrysiakŁukasz Pajchel


